# Supplementary material for: Integrating Complementary and Alternative Medicine into General Practice Training: A Regional Survey in South Tyrol
Source: Healthcare (Basel). 2025 Apr 2;13(7):797. doi: 10.3390/healthcare13070797 (PMC11988367; doi:10.3390/healthcare13070797)
Supplement: Supplementary file 1 [file healthcare-13-00797-s001.zip › healthcare-3541012-supplementary.pdf]

Supplementary Materials

File S1. Questionnaire on complementary and alternative medicine (CAM) in general practice training.

Introduction:
Complementary and alternative medicine (CAM) is becoming increasingly important, both in medical practice and in the public debate. In South Tyrol, an initial survey on the attitudes, experiences and applications of complementary medicine methods of general practitioners was conducted in 2017. This study now lays the foundation for an updated survey to analyze developments in this area, especially after the COVID-19 pandemic.

The aim of this questionnaire is to collect comprehensive information on attitudes, knowledge and the practical application of CAM and to enable comparisons with the results of the previous survey. The questionnaire is aimed at doctors who are training in general practice or have completed their training in the last 10 years and provides a valuable basis for better understanding the potential and challenges of CIM in the healthcare system.
This study is inspired by similar studies such as the needs assessment on CAM in specialist training in general practice, which shows that targeted training programs and the integration of CAM into medical practice are essential to improve patient care and meet the requirements of holistic medicine (Valentini et al. 2017).

Questionnaire:
This questionnaire consists of two parts: a general part (Likert scale) and a specific part (questions on methods and experiences). Please answer the questions carefully and according to the scale provided.

Likert scale:
0 = does not apply at all or never
5 = fully or very often applies

Checkbox questions:
Multiple answers are possible unless otherwise stated.

Part 1: General questions (Likert scale)
According to: Valentini J, Flum E, Schwill S, Krug K, Szecsenyi J, Joos S. Complementary and integrative medicine in residency training in general practice: results of a needs assessment of physicians in residency training. Complement Med Res. 2018;25(4):233-239. doi: 10.1159/000485319.

Questions:
1: I am interested in the topic of CIM.
2: The integration of CIM into GP care brings benefits for the patient.
3: For me, sufficient evidence is a prerequisite for the use of CIM.
4: CIM plays an important role in my daily work.
5: I actively ask my patients whether they use CIM themselves.
6: I am asked by my patients for advice on CIM.
7: I use CIM in my everyday medical practice.
8: I am already in the supplementary training phase for a complementary medical procedure.
9: I am aiming to obtain additional training in complementary medicine.
10: I use CIM on myself.
11: I consider my knowledge in the field of CIM to be sufficient.
12: I feel confident when patients ask me about CIM.
13: I feel confident in assessing possible interactions, side effects and contraindications in CIM.
14: I feel confident in my assessment of the evidence base for CIM.
15: I feel confident in assessing information in the (lay) press about CIM.
16: I feel confident when it comes to the assumption of costs for CIM.
17: I feel confident in matters relating to the legal regulations of the CIM.
18: Further training as a general practitioner should impart skills from the field of CIM.
19: I am interested in a course on CIM in the Verbundweiterbildung Plus.
20: I have already attended courses on complementary medicine topics as part of my further training.

Part 2: Questions about specific methods and experiences
According to: Marcher B. What do South Tyrolean general practitioners think about complementary medicine? A survey among conventional general practitioners in the health districts of Bressanone and Brunico. Final thesis as part of the special training for general practice 2014-2017. South Tyrolean Academy for General Practice, Bolzano, March 17, 2017

Questions:
1: Do you support any of the following complementary medicine methods?
- none
- Acupuncture
- Homeopathy
- Phytotherapy
- Manual therapy, osteopathy, chiropractic
- Neural therapy
- Folk medicine/domestic remedies
- Other (please specify):

2: Which complementary medicine method do you consider to be scientifically sound?
- none
- Acupuncture
- Homeopathy
- Phytotherapy
- Manual therapy, osteopathy, chiropractic
- Neural therapy
- Folk medicine/domestic remedies
- Other (please specify):

3: Which complementary medicine method have you had good experiences with?
- none
- Acupuncture
- Homeopathy
- Phytotherapy
- Manual therapy, osteopathy, chiropractic
- Neural therapy
- Folk medicine/domestic remedies
- Other (please specify):

4: Which complementary medicine method do you often recommend to your patients?
- none
- Acupuncture
- Homeopathy
- Phytotherapy
- Manual therapy, osteopathy, chiropractic
- Neural therapy
- Folk medicine/domestic remedies
- Other (please specify):

5: Which complementary medicine method do you use for yourself or your family?
- none
- Acupuncture
- Homeopathy
- Phytotherapy
- Manual therapy, osteopathy, chiropractic
- Neural therapy
- Folk medicine/domestic remedies
- Other (please specify):

6: For which complaints do you prescribe KM?
- Headache
- Musculoskeletal complaints
- Psychosomatic complaints
- Asthenia, weakness, fatigue
- Unclear complaints without disease value
- Pruritus sine materia
- Tinnitus, ears closed, etc.
- Irritable bowel syndrome etc.
- Colds, flu-like infections
- Other (please specify):

7: In which situation do you recommend complementary medicine methods?
- Never
- Instead of conventional medicine
- Complementary to conventional medicine
- At the patient's request
- As a placebo
- Other:

8: Have you learned a complementary medicine method yourself?
- No
- No, but I would like to learn
- I am currently learning
- Yes; which:

9: Do you offer KM in your practice?
- No, no interest
- No, no time, or other reasons
- Would I like to
- Yes; which:

10: Do you think it's enough for a GP to be able to refer their patients to good CM therapists?
- Yes.
- No, he should offer one or a few methods himself.
- No, a GP should not prescribe complementary medicine.

11: What do you think of your knowledge of KM?
- Outstanding
- Good
- Low
- Not sufficient

12:Do you find training for GPs on CM useful?
- Many
- Some
- None

Question 13: Do you think the KM should be financially supported by the public sector?
- Yes
- Mostly
- Rare
- Never

Table S1. Comparison of CAM-related attitudes, confidence, and practices across demographic and practice characteristics (*n* = 37).

Variable	Character-istic	Group	*n*	Mean (SD)	Median (IQR)	Test Statistic [1]	*p*-value	Effect Size [2]
I am interested in the topic of CAM	Sex	Female	24	2.75 (1.94)	3.00 (3.25)	184.0	0.375	0.18
		Male	13	2.15 (1.46)	2.00 (2.00)			
	Mother tongue	German	17	2.12 (1.65)	2.00 (2.00)	3.408	0.074	0.09
		Italian	19	2.84 (1.89)	3.00 (3.50)			
		Ladin	1	4.00 (nan)	4.00 (0.00)			
	Country of medicine studies	Austria	13	2.38 (1.61)	2.00 (3.00)	0.244	0.785	0.01
		Germany	5	2.20 (2.17)	1.00 (3.00)			
		Italy	19	2.74 (1.88)	3.00 (3.50)			
	Practice location	Urban	19	2.84 (1.83)	3.00 (3.00)	73.0	0.316	-0.23
		Rural	10	2.10 (1.91)	1.00 (3.00)			
The integration of CAM into primary care benefits patients	Sex	Female	24	3.04 (1.88)	3.50 (4.00)	205.0	0.116	0.31
		Male	13	2.00 (1.73)	2.00 (2.00)			
	Mother tongue	German	17	2.65 (1.69)	3.00 (4.00)	0.098	0.756	0.00
		Italian	19	2.84 (2.00)	3.00 (3.50)			
		Ladin	1	0.00 (---)[3]	0.00 (0.00)			
	Country of medicine studies	Austria	13	2.31 (1.55)	2.00 (2.00)	1.083	0.350	0.06
		Germany	5	2.00 (2.34)	1.00 (4.00)			
		Italy	19	3.10 (1.94)	4.00 (3.50)			
	Practice location	Urban	19	2.68 (1.92)	3.00 (3.50)	100.0	0.833	0.05
		Rural	10	2.80 (2.20)	3.00 (4.00)			
Adequate evidence is a prerequisite for applying CAM	Sex	Female	24	4.83 (1.18)	4.00 (1.00)	183.0	0.371	0.17
		Male	13	3.54 (1.61)	3.00 (2.00)			
	Mother tongue	German	17	3.88 (1.32)	4.00 (2.00)	0.008	0.931	0.00
		Italian	19	3.84 (1.42)	4.00 (2.00)			
		Ladin	1	5.00 (---)[3]	5.00 (0.00)			
	Country of medicine studies	Austria	13	4.15 (1.21)	5.00 (1.00)	0.403	0.672	0.02
		Germany	5	3.60 (1.67)	4.00 (2.00)			
		Italy	19	3.78 (1.40)	4.00 (2.00)			
	Practice location	Urban	19	3.90 (1.45)	5.00 (2.00)	88.0	0.749	-0.07
		Rural	10	3.80 (1.32)	4.00 (2.00)			
CAM plays an important role in my daily work	Sex	Female	24	1.92 (1.69)	2.00 (3.00)	165.0	0.782	0.06
		Male	13	1.69 (1.18)	2.00 (1.00)			
	Mother tongue	German	17	1.29 (1.45)	3.00 (2.00)	3.913	0.056	0.10
		Italian	19	2.26 (1.48)	1.00 (1.50)			
		Ladin	1	3.00 (---)[3]	3.00 (0.00)			
	Country of medicine studies	Austria	13	1.69 (1.55)	1.00 (1.00)	0.786	0.464	0.04
		Germany	5	1.20 (1.30)	1.00 (2.00)			
		Italy	19	2.10 (1.56)	2.00 (2.50)			
	Practice location	Urban	19	2.21 (1.69)	2.00 (2.50)	71.0	0.270	-0.25
		Rural	10	1.50 (1.35)	1.50 (1.75)			
I actively ask my patients whether they use CAM	Sex	Female	24	2.21 (1.69)	2.50 (2.00)	218.0	0.045	0.40
	Sex	Male	13	1.08 (1.19)	1.00 (2.00)			
	Mother tongue	German	17	1.82 (1.42)	1.00 (3.00)	0.004	0.951	0.00
		Italian	19	1.79 (1.84)	1.00 (2.50)			
		Ladin	1	2.00 (---)[3]	2.00 (0.00)			
	Country of medicine studies	Austria	13	1.78 (1.36)	2.00 (1.00)	0.479	0.623	0.03
		Germany	5	1.20 (1.30)	1.00 (2.00)			
		Italy	19	2.00 (1.86)	3.00 (3.00)			
	Practice location	Urban	19	1.90 (1.91)	1.00 (3.00)	82.5	0.568	-0.13
		Rural	10	1.40 (1.35)	1.50 (2.75)			
My patients ask me for advice regarding CAM	Sex	Female	24	2.79 (1.59)	2.00 (2.25)	164.0	0.499	0.14
		Male	12	2.42 (1.16)	2.00 (1.50)			
	Mother tongue	German	17	2.35 (1.37)	2.00 (2.00)	1.729	0.198	0.05
		Italian	18	3.00 (1.53)	4.00 (2.00)			
		Ladin	1	2.00 (---)[3]	2.00 (0.00)			
		Austria	13	2.15 (1.24)	2.00 (1.00)	1.327	0.279	0.07

	Country of medicine studies	Germany	5	2.80 (1.64)	2.00 (2.0)			
		Italy	18	3.00 (1.53)	4.00 (2.00)			
	Practice location	Urban	18	3.06 (1.55)	4.00 (2.75)	74.0	0.444	-0.18
		Rural	10	2.50 (1.18)	2.00 (0.75)			
I apply CAM in my medical practice	Sex	Female	24	2.04 (1.63)	2.00 (2.00)	206.5	0.103	0.32
		Male	13	1.15 (1.34)	1.00 (2.00)			
	Mother tongue	German	17	1.59 (1.33)	2.00 (1.00)	1.463	0.501	0.01
		Italian	19	1.95 (1.78)	2.00 (3.00)			
		Ladin	1	0.00 (---)[3]	0.00 (0.00)			
	Country of medicine studies	Austria	13	1.38 (0.95)	1.00 (1.00)	1.993	0.152	0.10
		Germany	5	1.00 (1.42)	0.00 (2.00)			
		Italy	19	2.00 (1.84)	2.00 (3.00)			
	Practice location	Urban	19	1.79 (1.75)	2.00 (3.00)	94.5	1.000	0.00
		Rural	10	1.70 (1.42)	2.00 (2.50)			
I am currently undergoing additional training in a CAM method	Sex	Female	24	1.33 (2.06)	0.00 (2.50)	NaN[a]		
		Male	12	0.00 (0.00)	0.00 (0.00)			
	Mother tongue	German	17	0.41 (1.28)	0.00 (0.00)	2.690	0.110	0.08
		Italian	18	1.39 (2.12)	0.00 (2.00)			
		Ladin	1	0.00 (---)[3]	0.00 (0.00)			
	Country of medicine studies	Austria	13	0.15 (0.55)	0.00 (0.00)	2.384	0.108	0.13
		Germany	5	2.00 (2.74)	0.00 (5.00)			
		Italy	18	1.11 (1.94)	0.00 (1.50)			
	Practice location	Urban	18	1.50 (2.09)	0.00 (3.50)	66.0	0.157	-0.27
		Rural	10	0.50 (1.58)	0.00 (0.00)			
I plan to pursue additional training in CAM	Sex	Female	24	1.88 (2.13)	1.00 (4.00)	201.5	0.117	0.29
		Male	13	0.69 (1.44)	0.00 (1.00)			
	Mother tongue	German	17	0.94 (1.68)	0.00 (1.00)	2.342	0.135	0.06
		Italian	19	1.95 (2.20)	1.00 (4.00)			
		Ladin	1	1.00 (---)[3]	1.00 (0.00)			
	Country of medicine studies	Austria	13	0.84 (1.46)	0.00 (1.00)	1.261	0.296	0.07
		Germany	11	1.20 (2.17)	0.00 (1.00)			
		Italy	5	1.95 (2.20)	1.00 (4.00)			
	Practice location	Urban	19	1.90 (2.23)	0.00 (4.00)	85.0	0.637	-0.10
		Rural	10	1.10 (1.60)	0.50 (1.75)			
I use CAM personally	Sex	Female	24	1.75 (1.92)	1.00 (3.00)	181.0	0.417	0.16
		Male	13	1.23 (1.64)	1.00 (2.00)			
	Mother tongue	German	17	1.29 (1.76)	1.00 (2.00)	0.789	0.381	0.02
		Italian	19	1.84 (1.92)	1.00 3.00)			
		Ladin	1	1.00 (---)[3]	1.00 (0.00)			
	Country of medicine studies	Austria	13	1.00 (1.64)	1.00 (2.00)	0.523	0.598	0.03
		Germany	5	2.20 (2.59)	1.00 (5.00)			
		Italy	19	1.63 (1.77)	1.00 (2.50)			
	Practice location	Urban	19	2.05 (2.04)	1.00 (4.00)	77.5	0.420	-0.18
		Rural	10	1.40 (1.78)	1.00 (1.75)			
I consider my knowledge of CAM sufficient.	Sex	Female	24	1.75 (1.26)	1.50 (1.25)	221.0	0.117	0.29
		Male	13	1.00 (1.35)	1.00 (1.00)			
	Mother tongue	German	17	1.24 (1.15)	1.00 (0.00)	0.519	0.476	0.01
		Italian	19	1.53 (1.26)	1.00 (1.00)			
		Ladin	1	5.00 (---)[3]	5.00 (0.00)			
	Country of medicine studies	Austria	13	1.31 (1.38)	1.00 (0.00)	0.432	0.652	0.02
		Germany	5	1.20 (1.64)	1.00 (1.00)			
		Italy	19	1.68 (1.25)	2.00 (1.00)			
	Practice location	Urban	19	1.37 (1.26)	1.00 (1.50)	99.5	0.849	0.05
		Rural	10	1.70 (1.77)	1.00 (2.50)			
I feel confident when patients ask me about CAM	Sex	Female	24	2.08 (1.56)	2.00 (2.00)	165.5	0.770	0.06
		Male	13	1.84 (1.21)	2.00 (2.00)			
	Mother tongue	German	17	2.06 (1.52)	2.00 (2.00)	0.014	0.905	0.00
		Italian	19	2.00 (1.41)	2.00 (2.00)			

		Ladin	1	1.00 (---)[3]	1.00 (0.00)			
	Country of medicine studies	Austria	13	1.62 (1.39)	1.00 (1.00)	0.954	0.395	0.05
		Germany	5	2.60 (1.95)	4.00 (3.00)			
		Italy	9	2.10 (1.34)	2.00 (1.50)			
	Practice location	Urban	19	2.26 (1.48)	2.00 (1.50)	102.0	0.761	0.07
		Rural	10	2.40 (1.43)	2.50 (2.50)			
I feel confident evaluating potential interactions, side effects, and contra-indications in CAM	Sex	Female	24	2.08 (1.56)	2.00 (2.00)	181.5	0.415	0.16
		Male	13	1.62 (1.19)	2.00 (1.00)			
	Mother tongue	German	17	1.88 (1.45)	2.00 (2.00)	0.46	0.795	0.02
		Italian	19	2.00 (1.49)	2.00 (2.00)			
		Ladin	1	1.00 (---)[3]	1.00 (0.00)			
	Country of medicine studies	Austria	13	1.54 (1.51)	1.00 (1.00)	0.711	0.498	0.04
		Germany	5	2.00 (1.41)	3.00 (2.00)			
		Italy	19	2.16 (1.42)	2.00 (1.50)			
	Practice location	Urban	19	2.37 (1.61)	2.00 (1.50)	76.5	0.396	-0.20
		Rural	10	1.80 (1.03)	2.00 (1.75)			
I feel confident evaluating the evidence base for CAM	Sex	Female	24	2.38 (1.74)	2.00 (3.00)	182.0	0.409	0.16
		Male	13	1.85 (1.34)	2.00 (2.00)			
	Mother tongue	German	17	2.23 (1.56)	2.00 (3.00)	0.057	0.812	0.00
		Italian	19	2.13 (1.73)	2.00 (2.00)			
		Ladin	1	3.00 (---)[3]	3.00 (0.00)			
	Country of medicine studies	Austria	13	1.92 (1.60)	1.00 (2.00)	0.056	0.815	0.00
		Germany	11	2.80 (1.64)	3.00 (1.00)			
		Italy	5	2.21 (1.65)	2.00 (2.00)			
	Practice location	Urban	19	2.53 (1.78)	2.00 (3.00)	82.5	0.576	-0.13
		Rural	10	2.10 (1.52)	2.50 (2.00)			
I feel confident evaluating CAM-related information in lay media	Sex	Female	24	2.08 (1.72)	1.50 (2.00)	177.0	0.504	0.14
		Male	13	1.62 (1.39)	1.00 (1.00)			
	Mother tongue	German	17	1.82 (1.59)	1.00 (2.00)	0.051	0.882	0.00
		Italian	19	1.95 (1.68)	2.00 (2.50)			
		Ladin	1	3.00 (---)[3]	3.00 (0.00)			
	Country of medicine studies	Austria	13	1.69 (1.38)	1.00 (2.00)	1.351	0.272	0.07
		Germany	5	3.00 (1.58)	3.00 (2.00)			
		Italy	19	1.79 (1.72)	1.00 (3.00)			
	Practice location	Urban	19	2.10 (1.63)	2.00 (2.00)	90.5	0.852	-.05
		Rural	10	2.00 (1.76)	2.00 (2.75)			
I feel confident regarding CAM reimbursement questions	Sex	Female	24	1.71 (1.76)	1.00 (3.00)	153.5	0.756	0.07
		Male	12	1.33 (1.16)	1.00 (1.25)			
	Mother tongue	German	17	1.24 (1.35)	1.00 (2.00)	1.488	0.231	0.04
		Italian	19	1.89 (1.78)	1.50 (2.75)			
		Ladin	1	2.00 (---)[3]	2.00 (0.00)			
	Country of medicine studies	Austria	13	1.31 (1.82)	1.00 (2.00)	1.489	0.240	0.08
		Germany	5	0.80 (1.30)	0.00 (1.00)			
		Italy	18	2.00 (1.71)	2.00 (2.00)			
	Practice location	Urban	18	2.00 (1.72)	2.00 (2.00)	68.0	0.290	-0.24
		Rural	10	1.30 (1.57)	0.50 (2.75)			
I feel confident regarding CAM legal regulations	Sex	Female	24	1.33 (1.52)	1.00 (2.00)	149.0	0.829	-0.04
		Male	13	1.38 (1.39)	1.00 (2.00)			
	Mother tongue	German	17	1.18 (1.41)	0.00 (2.00)	0.684	0.4144	0.02
		Italian	19	1.53 (1.54)	1.00 (2.50)			
		Ladin	1	2.00 (---)[3]	2.00 (0.00)			
	Country of medicine studies	Austria	13	1.54 (1.45)	1.00 (2.00)	0.820	0.449	0.05
		Germany	5	0.80 (1.30)	0.00 (1.00)			
		Italy	19	1.63 (1.45)	1.00 (1.50)			
	Practice location	Urban	19	1.68 (1.53)	1.00 (2.00)	74.0	0.332	-0.22
		Rural	10	1.10 (1.29)	0.50 (2.00)			
GP training should include	Sex	Female	24	2.92 (1.98)	4.00 (4.00)	193.0	0.237	0.24
		Male	13	2.31 (1.64)	2.00 (2.00)			
		German	17	2.76 (1.68)	3.00 (2.00)	0.002	0.965	0.00

competencies in CAM	Mother tongue	Italian	19	2.74 (2.02)	3.00 (4.00)			
		Ladin	1	0.00 (---)[3]	0.00 (0.00)			
	Country of medicine studies	Austria	13	2.69 (1.75)	2.00 (2.00)	1.486	0.241	0.08
		Germany	5	1.40 (1.52)	1.00 (3.00)			
		Italy	19	3.00 (1.97)	4.00 (3.50)			
	Practice location	Urban	19	2.58 (1.95)	3.00 (3.5)	88.5	0.779	-0.07
		Rural	10	2.40 (1.71)	2.50 (2.50)			
I am interested in a CAM course in the GP training program	Sex	Female	24	2.83 (1.86)	3.00 (2.50)	204.5	0.120	0.31
		Male	13	1.85 (1.68)	2.00 (3.00)			
	Mother tongue	German	17	2.06 (1.71)	2.00 (3.00)	1.427	0.240	0.04
		Italian	19	2.79 (1.93)	3.00 (3.50)			
		Ladin	1	4.00 (---)[3]	4.00 (0.00)			
	Country of medicine studies	Austria	13	2.08 (1.75)	2.00 (3.00)	0.496	0.613	0.03
		Germany	5	2.60 (1.82)	3.00 (1.00)			
		Italy	19	2.74 (1.94)	3.00 (3.50)			
	Practice location	Urban	19	2.78 (1.87)	3.00 (3.00)	78.0	0.440	-0.18
		Rural	10	2.20 (1.87)	2.50 (3.50)			
I have already attended CAM-related courses as part of GP training of GPs	Sex	Female	24	1.83 (2.14)	0.50 (4.00)	166.5	0.731	0.07
		Male	13	1.54 (2.02)	0.00 (3.00)			
	Mother tongue	German	17	1.00 (1.54)	0.00 (2.00)	3.408	0.074	0.09
		Italian	19	2.21 (2.78)	2.00 (4.50)			
		Ladin	1	5.00 (---)[3]	5.00 (0.00)			
	Country of medicine studies	Austria	13	1.38 (1.98)	0.00 (2.00)	0.458	0.636	0.03
		Germany	5	1.40 (2.19)	0.00 (2.00)			
		Italy	19	2.05 (2.17)	2.00 (4.00)			
	Practice location	Urban	19	2.10 (2.13)	2.00 (4.00)	73.0	0.293	-0.23
		Rural	10	1.20 (1.87)	0.00 (1.75)			

[1] Mann-Whitney U tests were employed for two-group comparisons (sex, practice location), Kruskal-Wallis H tests were used for comparisons among three groups (mother tongue, country of medical studies); H and U values are given. [2] Effect sizes were calculated using Rank-Biserial Correlation (r) for Mann-Whitney U tests and interpreted as small ($r < 0.3$), moderate ($r = 0.3–0.5$), or large ($r > 0.5$), and positive values indicate that the first group (e.g., females) had higher ranks than the second (e.g., males), whereas negative values indicate that the second group had higher ranks than the first; Eta-Squared (η^2) was calculated for Kruskal-Wallis H tests and interpreted as small ($\eta^2 < 0.10$), medium ($\eta^2 = 0.10–0.30$), or large ($\eta^2 > 0.30$) (Lenhard & Lenhard, 2022); r and η^2 values are given. [3] Missing due to $n = 1$. [a]The variance i is equal to 0 after grouping on Sex. Abbreviations: CAM, complementary and alternative medicine; GP, general practitioner; IQR, interquartile range; SD, standard deviation.
